# Integrative Multi-Omics and Machine Learning Identify ID1 as a Candidate Gene Associated with Abdominal Aortic Aneurysm

**DOI:** 10.3390/cimb48020156

**Published:** 2026-01-30

**Authors:** Feng Guo, Michael Keese, Yu Zhao, Qining Fu

**Affiliations:** 1 Department of Vascular Surgery, The First Affiliated Hospital of Chongqing Medical University, Chongqing 400016, China; guofengsc12@gmail.com (F.G.); zhao_yu_cqmu_1965@163.com (Y.Z.); 2 Department of Vascular Surgery, Theresienkrankenhaus, 68165 Mannheim, Germany; m.keese@theresienkrankenhaus.de

**Keywords:** abdominal aortic aneurysm, ID1, machine learning, immune microenvironment, WGCNA, single-cell RNA sequencing

## Abstract

Abdominal aortic aneurysm (AAA) is a fatal vascular disorder driven by immune dysregulation and extracellular matrix (ECM) degradation, yet its molecular mechanisms remain unclear. This study investigated the mechanistic role of ID1 in AAA using an integrative multi-omics and machine learning approach. Two bulk transcriptomic datasets (GSE232911 and GSE183464) were analyzed through differential expression, WGCNA, and three machine learning algorithms (LASSO, Random Forest, and SVM-RFE), followed by immune infiltration analysis via ssGSEA and CIBERSORT. ID1 and CYP4B1 were identified by all three machine learning algorithms, but only ID1 showed stable downregulation and consistent discriminatory ability across independent datasets. (AUC = 0.939 and 0.868). Functional enrichment and immune deconvolution linked low ID1 expression to enhanced adaptive immune signaling, increased M1 macrophages, γδ T cells, and memory B cells, and reduced neutrophil and mast cell activity. Single-cell RNA sequencing (GSE226492) confirmed endothelial- and fibroblast-specific ID1 downregulation in AAA. These findings identify ID1 as a candidate gene associated with vascular immune remodeling and extracellular matrix–related pathways, providing a basis for future mechanistic investigation in AAA.

## 1. Introduction

Abdominal aortic aneurysm (AAA) is a chronic degenerative vascular disorder characterized by irreversible, progressive dilation of the abdominal aorta [1,2]. It predominantly affects elderly males, smokers, and patients with hypertension [3]. The disease is usually asymptomatic in its early stages and progresses slowly with gradual expansion of the aortic diameter. A meta-analysis by Sweeting et al. [4] estimated an average growth rate of approximately 2.2 mm per year, influenced by baseline diameter, smoking, hypertension, and female sex. The risk of rupture increases markedly with aneurysm size and is particularly high in women and smokers. Once rupture occurs, the mortality rate exceeds 80% [5,6]. Epidemiological data indicate that the prevalence of AAA in men over 65 years ranges from 4 to 8% [3,7]. Given that aneurysms larger than 5.5 cm carry a particularly high rupture risk, understanding disease mechanisms and identifying molecular determinants of AAA progression are critical to improving patient outcomes.

Currently, imaging modalities such as ultrasound, computed tomography (CT), and magnetic resonance imaging (MRI) are routinely used for AAA screening and surveillance. Aneurysm diameter remains the main clinical indicator [8,9,10], though it varies across imaging techniques and does not fully reflect the underlying biological processes. Recent studies have suggested that features such as intraluminal thrombus formation and mural hemorrhage are also linked to rupture risk [9], underscoring the need for molecular-level insights beyond structural metrics alone.

At the molecular level, AAA development involves not only atherosclerosis but also chronic inflammation, immune dysregulation, vascular smooth muscle cell apoptosis, and extracellular matrix (ECM) degradation [11,12,13]. These mechanisms interact in a complex manner, driving both vascular remodeling and progressive wall weakening. Although this growing mechanistic understanding provides a foundation for identifying disease-associated genes, clinically applicable molecular indicators for assessing AAA activity remain limited. Advances in high-throughput sequencing, including bulk RNA sequencing (RNA-seq) and single-cell RNA sequencing (scRNA-seq), now enable detailed characterization of the molecular and cellular landscape of AAA [14].

Bioinformatics and systems biology tools have become essential for deciphering these data. Weighted gene co-expression network analysis (WGCNA) can identify co-regulated gene modules related to disease phenotypes [15]. When integrated with machine learning algorithms such as Least Absolute Shrinkage and Selection Operator (LASSO), Random Forest (RF), and Support Vector Machine–Recursive Feature Elimination (SVM-RFE), these approaches enable the discovery of robust and biologically meaningful gene signatures [16]. Moreover, immune deconvolution algorithms such as CIBERSORT allow for quantification of immune cell subsets within bulk transcriptomic data [17,18], while scRNA-seq provides single-cell resolution to delineate the cellular origins and functional states of key regulators [19,20]. Despite these advances, the combined application of multi-omics integration, immune profiling, and machine learning to investigate AAA pathogenesis remains underexplored.

In this study, we implemented an integrative multi-omics framework combining bulk RNA-seq, scRNA-seq, and immune infiltration analyses to elucidate the molecular and immunological mechanisms underlying AAA. Rather than aiming to establish a clinical diagnostic biomarker, our objective was to identify key regulatory genes—particularly ID1—and explore their mechanistic relevance to vascular inflammation and immune remodeling. This approach provides new insights into AAA pathobiology and potential molecular targets for future mechanistic and translational studies.

## 2. Materials and Methods

### 2.1. Data Acquisition and Preprocessing

The microarray dataset GSE232911 (platform: GPL17586, Affymetrix Human Transcriptome Array 2.0; Affymetrix, Santa Clara, CA, USA) and the bulk RNA-sequencing dataset GSE183464 (platform: GPL20301, Illumina HiSeq 4000; Illumina Inc., San Diego, CA, USA) were downloaded from the Gene Expression Omnibus (GEO) database [21] on 27 March 2025. Both datasets contain fully anonymized gene-expression profiles of human abdominal aortic tissues from patients with AAA and non-aneurysmal controls; no personally identifiable information was accessed.

For GSE232911, raw CEL files were imported and background-corrected using the Robust Multi-array Average (RMA) method implemented in the oligo (v1.64.0) package. For GSE183464, raw count data were normalized and log_2_-transformed to transcripts per million (TPM) using edgeR (v3.42.4) and limma (v3.56.2). Platform-specific annotation tables were applied to map probe IDs or Ensembl IDs to official gene symbols; when multiple probes corresponded to the same gene, the arithmetic mean of their expression values was used.

To reduce inter-platform bias, both datasets were quantile-normalized with the preprocessCore (v1.60.2) package, followed by z-score standardization across samples. Batch effects between datasets were evaluated by principal-component analysis (PCA) and corrected using the ComBat function in sva (v3.46.0). The resulting harmonized expression matrix was used for downstream analyses, including differential expression and co-expression network construction. Clinical variables such as age, sex, and smoking status were not consistently available across the public datasets and were therefore not incorporated into the current analytical framework.

### 2.2. Differential Expression Analysis

Differentially expressed genes (DEGs) between AAA and control samples were identified using the limma package (v3.56.2) [22]. For the microarray dataset (GSE232911), probe-level data were background-corrected and normalized via the RMA algorithm. For the RNA-seq dataset (GSE183464), voom transformation was applied to convert count data into log_2_-counts per million (logCPM) with precision weights. Linear models were fitted for each gene, and Empirical Bayes moderation was used to estimate moderated t-statistics and log_2_ fold changes. The Benjamini–Hochberg method was applied to control the false discovery rate (FDR). Genes with adjusted *p*-values < 0.05 and |log_2_ fold change| > 1 were considered significantly differentially expressed. Volcano and heatmap plots were generated using ggplot2 (v3.5.1) and pheatmap (v1.0.12), respectively. The voom–limma framework was selected to ensure methodological consistency across microarray and RNA-seq platforms and compatibility with downstream WGCNA and machine learning analyses.

### 2.3. WGCNA

WGCNA was performed using the WGCNA package (v1.72.1, The WGCNA Team, Harvard University, Cambridge, MA, USA) [23] to identify gene co-expression modules associated with the AAA phenotype. To reduce noise, genes were ranked by expression variance across samples, and the top 25% most variable genes were retained for network construction. After quality control using the goodSamplesGenes function and outlier assessment by hierarchical clustering, a total of 8374 genes across all samples were used for WGCNA.

An appropriate soft-thresholding power was selected using the scale-free topology criterion, and a power of β = 14 was chosen for network construction (scale-free topology fit R^2^ > 0.85). Co-expression modules were identified using the blockwiseModules function with an unsigned topological overlap matrix (TOMType = “unsigned”), a minimum module size of 30, and module eigengenes were merged using a mergeCutHeight of 0.25 (corresponding to an eigengene correlation > 0.75). In total, 17 distinct co-expression modules (excluding the gray module) were identified. Module–trait relationships were evaluated by Pearson correlation between module eigengenes and the AAA phenotype, and the module showing the strongest association with AAA was selected for downstream analyses.

### 2.4. Feature Selection Using Machine Learning

To prioritize key genes associated with AAA, three complementary machine learning algorithms were implemented using glmnet (v4.1-8), randomForest (v4.7-1.2), and e1071 (v1.7-14) packages in R:

1. LASSO regression with 10-fold cross-validation was applied to the intersection of DEGs and AAA-associated WGCNA module genes to prevent overfitting. The optimal penalty parameter (λ) was determined based on the minimum cross-validation error [24].
2. RF classification was performed with 500 trees, and feature importance was ranked using the MeanDecreaseGini index. Model performance was evaluated using out-of-bag (OOB) error and receiver operating characteristic (ROC) curves [25].
3. SVM-RFE was conducted using a linear kernel and 10-fold cross-validation to iteratively eliminate the least informative features and identify genes with maximal classification accuracy [26].

Genes consistently selected by all three algorithms were retained as robust AAA-related candidates for downstream analysis. Data partitioning and cross-validation were conducted within each dataset to avoid information leakage and ensure generalizability.

### 2.5. Immune Function and Infiltration Analysis

Immune-related functional enrichment was evaluated using single-sample gene set enrichment analysis (ssGSEA) implemented in the GSVA package (v1.48.2) [27], based on 22 predefined immune cell and pathway signatures. Immune cell infiltration was estimated by the CIBERSORT algorithm (v1.04) [28] using the LM22 leukocyte signature matrix (developed by Newman et al., Stanford University, Stanford, CA, USA) with 1000 permutations. Only samples with deconvolution *p*-values < 0.05 were retained for subsequent analysis. Comparisons of immune infiltration between high- and low-ID1 expression groups (stratified by median expression) were performed using the Wilcoxon rank-sum test. *p*-values were adjusted by the Benjamini–Hochberg method, and visualization was performed with ggplot2.

### 2.6. Correlation Analysis

Spearman’s rank correlation analysis was used to assess associations between ID1 expression levels and immune cell proportions estimated from CIBERSORT. Correlation coefficients (r) and corresponding *p*-values were calculated using the Hmisc package (v5.1-3). Statistically significant associations (adjusted *p* < 0.05) were visualized using scatter plots and summarized as bubble plots generated by ggplot2 (v3.5.1), where color indicated correlation direction and dot size represented correlation strength.

### 2.7. scRNA-Seq Validation

The scRNA-seq dataset GSE226492 was downloaded from GEO and processed using Seurat (v4.3.0, CRAN/Satija Lab, New York, NY, USA) [29]. Quality control was applied to retain cells with 200–2500 detected genes, <10% mitochondrial transcripts, and >1000 unique molecular identifiers (UMIs). Gene expression values were log-normalized, and data were scaled prior to dimensionality reduction via PCA. The top 20 principal components were used for clustering using the shared nearest neighbor (SNN) modularity optimization algorithm with a resolution of 0.8. Cell type annotation was performed by reference to canonical marker genes. Two-dimensional visualization was achieved using t-distributed stochastic neighbor embedding (t-SNE). ID1 expression was projected onto cell clusters, and statistical differences between AAA and control samples were assessed using the Wilcoxon rank-sum test (*p* < 0.05).

## 3. Results

### 3.1. Integrative Identification of AAA-Associated Genes via Differential and Co-Expression Network Analysis

**Figure 1:**
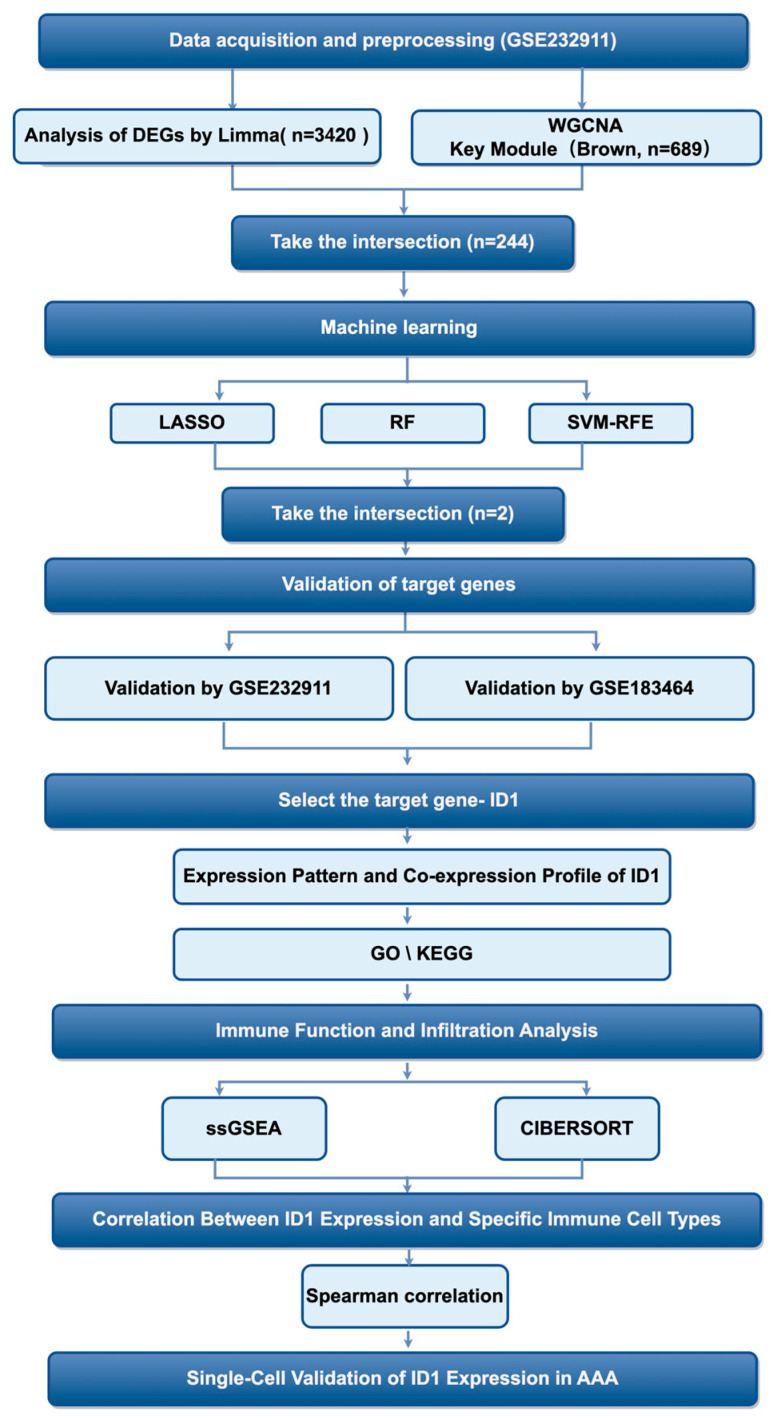
Diagram showing the sequence of steps conducted in this research.

First of all, the analytical workflow is summarized in Figure 1. The investigation began with analysis of the GSE232911 dataset from GEO, which contains microarray-based transcriptomic profiles of abdominal aortic tissue samples from patients with AAA and non-aneurysmal controls. Raw expression data were preprocessed and normalized to ensure cross-sample comparability and technical consistency for downstream analyses.

**Figure 2:**
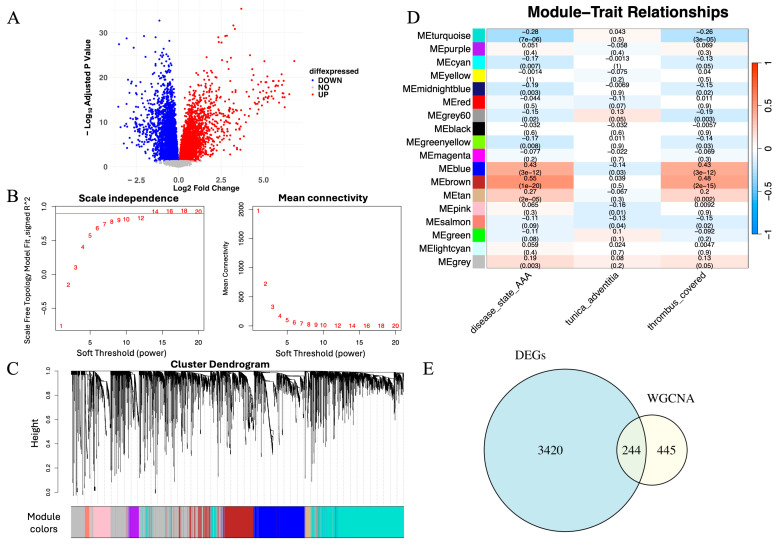
Integrative identification of genes associated with AAA through differential expression and co-expression network analysis. (**A**) Volcano plot showing DEGs between AAA and control samples (adjusted *p* < 0.05, |log_2_FC| > 1). Red and blue dots represent upregulated and downregulated genes, respectively. (**B**) Scale-free topology and mean connectivity plotted against soft-thresholding powers in WGCNA. The optimal power (β = 14) was selected for scale-free topology. The red line represents the cutoff for determining the appropriate soft-thresholding power based on scale-free topology fit (R^2^ = 0.9). (**C**) Cluster dendrogram of samples used to detect outliers, with modules identified based on hierarchical clustering. The red line represents the cutoff for identifying outlier samples, and samples to the right were removed. (**D**) Module–trait correlation heatmap showing the brown module’s positive association with the AAA phenotype (correlation = 0.81, *p* < 0.01). (**E**) Venn diagram depicting 244 overlapping genes between DEGs and those within the brown module, representing high-confidence AAA-associated candidates.

To identify genes potentially involved in AAA pathogenesis, we performed differential expression analysis using the limma package. Genes with adjusted *p* < 0.05 and |log_2_FC| > 1 were considered significant. A total of 3420 DEGs were detected, comprising 1802 upregulated and 1618 downregulated genes. A volcano plot illustrates the global distribution of differential expression (Figure 2A), and a heatmap highlights distinct transcriptional patterns between AAA and control samples (Figure 2B), reflecting marked molecular divergence between the two groups.

To investigate gene regulation at the systems level, we applied WGCNA. Based on the scale-free topology criterion, a soft-thresholding power of β = 14 was selected to construct a scale-free network (Figure 2C). Hierarchical clustering identified multiple co-expression modules, among which the brown module exhibited the strongest positive correlation with the AAA phenotype (correlation = 0.81, *p* < 0.01; Figure 2D). WGCNA identified a total of 17 co-expression modules (excluding the gray module), among which several showed weaker positive or negative correlations with the AAA phenotype. The brown module exhibited the strongest positive association with AAA (r = 0.81, *p* < 0.01) and was therefore selected for subsequent analyses.

To obtain high-confidence genes related to AAA, we intersected the DEGs with the brown module genes, yielding 244 overlapping genes (Figure 2E). These genes were both significantly dysregulated and embedded within an AAA-associated co-expression network, representing a robust set of candidates for subsequent machine learning–based prioritization and downstream analyses.

### 3.2. Machine Learning-Based Identification of Robust AAA-Associated Candidate Genes

To further prioritize candidate genes associated with AAA, we applied three complementary machine learning algorithms—LASSO regression, RF, and SVM-RFE—to the 244 high-confidence genes obtained from the integrated DEG and WGCNA analyses.

**Figure 3:**
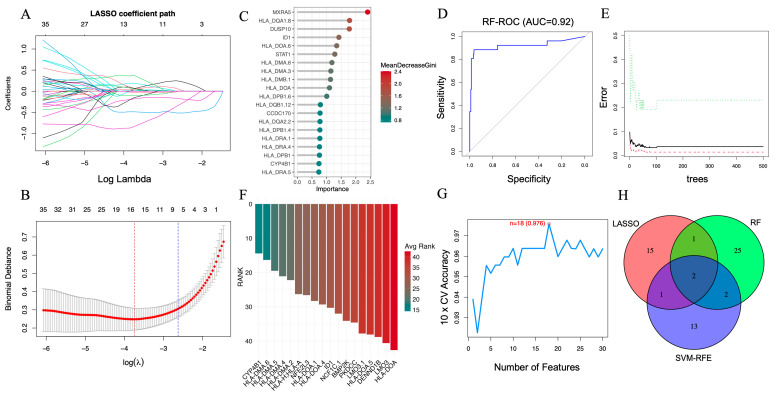
Screening of key AAA-associated genes using machine learning algorithms. (**A**) LASSO coefficient profile illustrating the selection of candidate genes. The x-axis shows log Lambda values, representing the regularization parameter, while the y-axis displays the LASSO coefficients. Each colored line represents a different gene, with color denoting specific genes and tracking their coefficient changes as log Lambda increases. (**B**) Ten-fold cross-validation curve identifying the optimal λ value for the LASSO model. (**C**) Variable importance ranking of the top 20 genes determined by the RF algorithm. (**D**) ROC curve of the RF classifier (AUC = 0.92). (**E**) Error rate of the RF model as the number of decision trees increases. (**F**) Feature-ranking curve generated by the SVM-RFE algorithm. (**G**) Cross-validation accuracy of the SVM-RFE model (peak accuracy = 0.976). (**H**) Venn diagram depicting ID1 and CYP4B1 as the two common genes consistently identified by LASSO (19), RF (30), and SVM-RFE (18), representing robust AAA-related candidates for further validation.

LASSO regression was implemented using 10-fold cross-validation to prevent overfitting and identify the optimal regularization parameter (λ). As shown in the coefficient trajectory plot (Figure 3A), increasing λ progressively reduced the coefficients of less informative genes toward zero. The minimum cross-validation error determined the optimal λ value (Figure 3B), yielding 19 genes with non-zero coefficients for subsequent evaluation.

In parallel, the RF algorithm was used to assess feature importance based on the MeanDecreaseGini index. The top 20 genes contributing most to classification accuracy are presented in Figure 3C. Model performance, evaluated using ROC analysis, achieved an area under the curve (AUC) of 0.92 (Figure 3D) with a stable error rate across an increasing number of decision trees (Figure 3E), indicating strong generalizability.

The SVM-RFE algorithm was then applied with a linear kernel and 10-fold cross-validation to iteratively rank features by predictive importance. Eighteen genes were selected based on the best-performing subset across resampling iterations (Figure 3F). The corresponding cross-validation curve showed a peak classification accuracy of 0.976 (Figure 3G), supporting the stability of the selected feature set.

To ensure robustness, we identified the intersection of genes retained by all three algorithms. As illustrated in Figure 3H, ID1 and CYP4B1 were consistently selected across all models, indicating their strong and reproducible association with AAA. These genes were subsequently evaluated in independent datasets and characterized using downstream transcriptomic and immune-related analyses.

### 3.3. Validation of Candidate Genes in Independent Datasets

**Figure 4:**
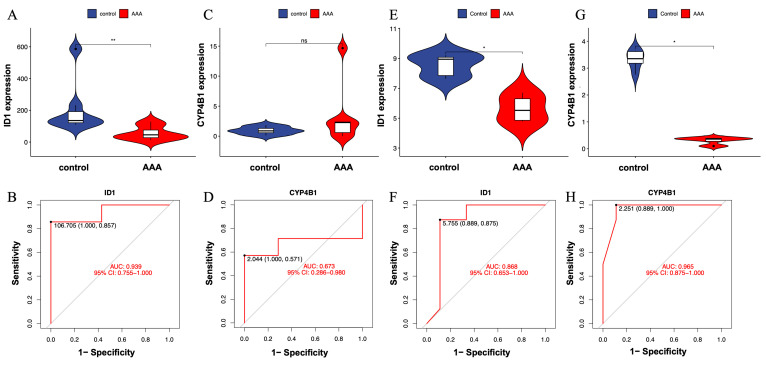
Validation of expression patterns for candidate genes ID1 and CYP4B1 in independent datasets. (**A**) Violin plot showing significantly lower expression of ID1 in AAA tissues compared with controls (*p* < 0.01). (**B**) ROC curve illustrating the classification performance of ID1 in the training dataset (GSE232911; AUC = 0.939). (**C**) Violin plot showing no significant difference in CYP4B1 expression between AAA and control groups (ns). (**D**) ROC curve for CYP4B1 in GSE232911 (AUC = 0.673). (**E**) Violin plot showing consistent downregulation of ID1 in the external validation dataset (GSE183464). (**F**) ROC curve of ID1 in GSE183464 (AUC = 0.868), confirming reproducible expression trends across datasets. (**G**) Violin plot showing significantly lower expression of CYP4B1 in AAA tissues in GSE183464 (*p* < 0.05). (**H**) ROC curve of CYP4B1 in GSE183464 (AUC = 0.965). Statistical significance is indicated as follows: * *p* < 0.05, ** *p* < 0.01, ns = not significant.

To evaluate the robustness and reproducibility of the two candidate genes—ID1 and CYP4B1—identified through integrative machine learning analysis, we first examined their expression levels and classification performance in the discovery dataset (GSE232911). As shown in Figure 4A, ID1 was significantly downregulated in AAA tissues compared with controls (*p* < 0.01), indicating a significant difference between AAA and control samples. Correspondingly, ROC analysis yielded an AUC of 0.939 (Figure 4B), indicating excellent model discrimination between AAA and control samples.

In contrast, CYP4B1 did not show a significant difference in expression between AAA and control samples in the same dataset (Figure 4C). However, its ROC curve exhibited moderate classification performance (AUC = 0.673; Figure 4D), indicating limited yet detectable separation ability despite the lack of significant differential expression.

To further assess consistency across datasets, both genes were validated in an independent external cohort (GSE183464), which included seven AAA and seven control aortic wall samples. In this validation set, ID1 was again significantly downregulated in AAA tissues (Figure 4E), with an AUC of 0.868 (Figure 4F), demonstrating reproducible expression patterns and consistent discriminative performance across datasets.

Interestingly, CYP4B1 showed no significant difference between AAA and control tissues in the discovery cohort (Figure 4C), but was significantly lower in AAA tissues in the validation cohort (Figure 4G). Despite this variation, its ROC analysis yielded a high AUC = 0.965 (Figure 4H), indicating strong discriminatory performance and indicating dataset-dependent discriminatory performance.

These results indicate that ID1 is a reproducibly dysregulated gene associated with AAA across independent datasets. CYP4B1 showed inconsistent statistical significance across datasets, suggesting that its role requires additional validation and should be interpreted with caution.

### 3.4. Expression Pattern and Co-Expression Profile of ID1

To further characterize transcriptomic features associated with ID1, a gene consistently identified across multiple machine learning models, we examined its transcriptomic associations within the GSE232911 dataset. Samples were stratified into ID1-high and ID1-low groups based on the median expression level, and differential expression analysis was performed to identify genes correlated with ID1 expression dynamics.

**Figure 5:**
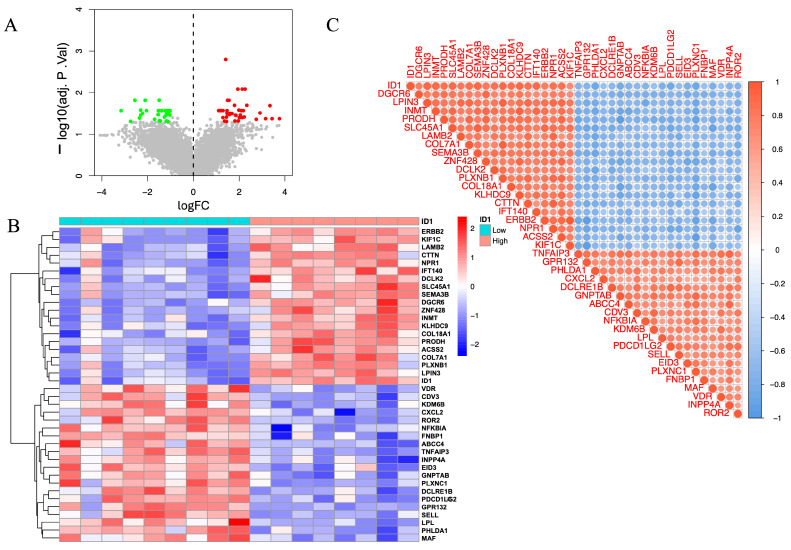
Expression profiling and co-expression analysis of ID1-associated genes in the GSE232911 dataset. (**A**) Volcano plot illustrating DEGs between ID1-high (|log_2_FC| > 1, adjusted *p* < 0.05, red) and ID1-low groups (log_2_FC < −1, adjusted *p* < 0.05, green). Grey points represent genes that are not significantly differentially expressed (|log_2_FC| ≤ 1 and adjusted *p* ≥ 0.05). (**B**) Heatmap displaying the top 20 upregulated and top 20 downregulated DEGs grouped according to ID1 expression levels. (**C**) Correlation matrix showing Pearson correlation coefficients between ID1 and selected DEGs, with red and blue representing positive and negative correlations, respectively.

The volcano plot (Figure 5A) illustrates widespread transcriptional alterations between ID1-low and ID1-high groups. A subset of genes was significantly upregulated in the ID1-high group (log_2_FC > 1, red), whereas others were enriched in the ID1-low group (log_2_FC < −1, green), based on an adjusted *p* < 0.05. These differences indicate distinct molecular programs associated with differential ID1 expression.

To visualize these trends, we generated a heatmap of the top 20 upregulated and top 20 downregulated genes (Figure 5B), which revealed a clear separation between ID1-high and ID1-low samples. Notably, genes such as ERBB2, KIF1C, LAMB2, CTTN, and NPR1 were enriched in the ID1-high group, whereas VDR, CDV3, KDM6B, CXCL2, and ROR2 were preferentially expressed in ID1-low samples. These genes are functionally linked to extracellular matrix organization, cellular adhesion, and inflammatory signaling, showing that these genes were enriched in ECM–related, cell adhesion–related, and immune/inflammatory transcriptional signatures.

We next explored co-expression relationships between ID1 and its associated DEGs using correlation matrix analysis (Figure 5C). Strong positive correlations were observed with genes including DEGCR6, LPIN3, INMT, PRODH, and SLC45A1, while negative correlations were detected with TNFAIP3, GPR132, PHLDA1, CXCL2, and DCLRE1B. These results indicate that ID1 is embedded in a broader co-expression network that includes genes related to metabolic processes, ECM organization, and inflammatory signaling.

### 3.5. Functional Enrichment Analysis of ID1-Associated Genes

To characterize biological processes and pathways associated with ID1-related gene expression, Gene Ontology (GO) and Kyoto Encyclopedia of Genes and Genomes (KEGG) enrichment analyses were performed on the DEGs associated with ID1 expression.

GO enrichment analysis revealed significant involvement of these genes in immune-related and structural biological processes. At the biological process level, the top enriched terms included the semaphorin–plexin signaling pathway, negative regulation of leukocyte proliferation, and chronic inflammatory response, suggesting that ID1 may participate in immune regulation and inflammatory control. In the cellular component category, enrichment of the semaphorin receptor complex and collagen-containing extracellular matrix indicated roles in extracellular signaling and vascular tissue remodeling. For molecular function, enrichment was observed in protease binding, extracellular matrix structural constituents, and semaphorin receptor activity (Supplementary Figure S1A).

To further elucidate gene–function relationships, a chord diagram was constructed to visualize gene–term associations (Supplementary Figure S1B). This analysis revealed strong interconnections among key DEGs involved in immune and inflammatory pathways, reinforcing the potential role of ID1-associated genes in AAA-related immune dysregulation.

KEGG pathway analysis identified several significantly enriched pathways, including Tuberculosis, Phagosome, Staphylococcus aureus infection, Lipid and atherosclerosis, and Osteoclast differentiation (Supplementary Figure S1C). These pathways are closely linked to inflammatory activation, macrophage function, and vascular remodeling—biological processes central to AAA progression. A circular KEGG interaction plot further illustrated the mapping of these DEGs to key signaling cascades (Supplementary Figure S1D).

Collectively, these enrichment results indicate that ID1-associated genes are enriched in immune-related, inflammatory, and ECM–related pathways in AAA.

### 3.6. Immune Functional Landscape Stratified by ID1 Expression

To investigate the immunological context associated with ID1 expression in AAA, we performed ssGSEA to evaluate the activity of 22 immune-related functional pathways. Samples were divided into high- and low-ID1 expression groups according to the median expression value.

**Figure 6:**
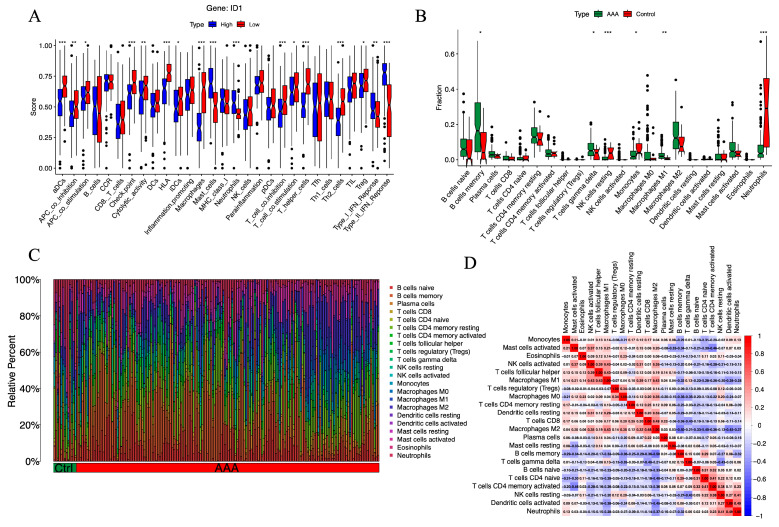
Immune functional activity and cellular infiltration landscape associated with ID1 expression in AAA. (**A**) Boxplots showing ssGSEA scores for 22 immune-related pathways in AAA samples stratified by ID1 expression level. Statistical significance is indicated as follows: * *p* < 0.05, ** *p* < 0.01, *** *p* < 0.001. (**B**) Stacked bar plot illustrating the relative abundance of 22 immune cell populations in AAA and control groups as estimated by CIBERSORT. Statistical significance is indicated as follows: * *p* < 0.05, ** *p* < 0.01, *** *p* < 0.001. (**C**) Boxplots comparing the proportions of individual immune cell types between the AAA and control groups. (**D**) Heatmap displaying Spearman correlation coefficients among immune cell subsets based on CIBERSORT-derived infiltration profiles.

As shown in Figure 6A, the low-ID1 group exhibited a distinct pattern of immune remodeling. The activities of several adaptive immune components were significantly enhanced, including activated dendritic cells (aDCs), immune checkpoint signaling, HLA expression, T cell co-inhibition, macrophage-associated responses, T helper cell activation, and Th2-type immunity (*p* < 0.001). Moderate increases were also observed in antigen-presenting cell (APC) co-inhibition and cytolytic activity, along with mild but notable elevations in regulatory T cells (Tregs), T cell co-stimulation, immature dendritic cells (iDCs), and APC co-stimulation (*p* < 0.05). In contrast, several innate immune components, such as mast cells, neutrophils, and type I and type II IFN signaling, were markedly suppressed (*p* < 0.001). These analyses revealed distinct immune functional activity patterns between ID1-high and ID1-low groups.

To further delineate the immune cell landscape, we employed CIBERSORT to estimate the relative proportions of 22 immune cell subsets. Compared with control samples, AAA tissues exhibited significantly reduced fractions of resting NK cells (*p* < 0.001), neutrophils (*p*< 0.001), and monocytes (*p* < 0.05), alongside increased infiltration of M1 macrophages (*p* < 0.01), γδ T cells, and memory B cells (*p* < 0.05) (Figure 6B,C). These alterations in immune composition are largely consistent with ssGSEA results, particularly the concordant increase in macrophage- and B cell–related immune functions in the low-ID1 group. Notably, although the overall proportion of neutrophils decreased, neutrophil-related functional scores were elevated, suggesting enhanced activation per cell or transient hyperactivity of specific subsets.

To explore immune–immune interactions, we constructed a correlation matrix of immune cell populations using Spearman coefficients (Figure 6D). Memory B cells were negatively correlated with M2 macrophages (r = −0.50), while activated mast cells were inversely related to activated CD4^+^ memory T cells (r = −0.46). In contrast, M2 macrophages showed a positive correlation with CD8^+^ T cells (r = 0.48), T follicular helper cells correlated with M1 macrophages (r = 0.43), and neutrophils were positively linked to activated dendritic cells (r = 0.49). These relationships suggest a coordinated immune interaction network influenced by ID1 expression, associated with an immune imbalance characteristic of AAA.

### 3.7. Correlation Between ID1 Expression and Specific Immune Cell Types

To further clarify the immunological relevance of ID1 in AAA, we performed Spearman correlation analysis between ID1 expression levels and the estimated proportions of 22 immune cell subsets inferred by CIBERSORT. This continuous-variable approach complements the previous group-based comparisons and provides a quantitative view of immune cell dynamics associated with ID1 expression.

**Figure 7:**
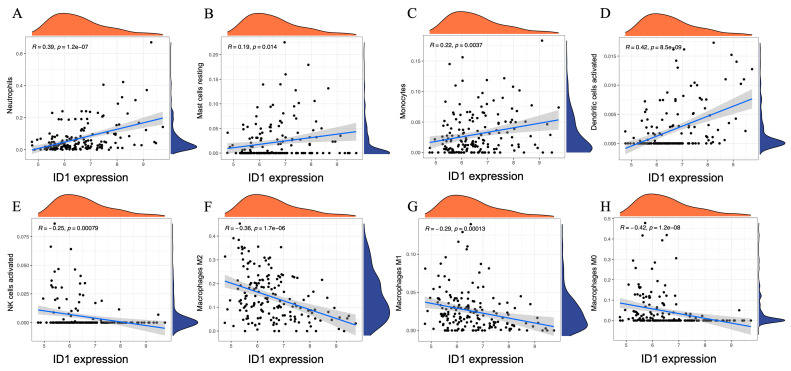
Spearman correlation between ID1 expression and immune cell proportions in AAA. (**A**–**H**) Scatter plots illustrating the Spearman correlation between ID1 expression levels and the estimated proportions of eight immune cell subsets in AAA samples, including (**A**) neutrophils, (**B**) resting mast cells, (**C**) monocytes, (**D**) activated dendritic cells, (**E**) activated NK cells, (**F**) M2 macrophages, (**G**) M1 macrophages, and (**H**) M0 macrophages. Immune cell fractions were inferred using the CIBERSORT algorithm. Each panel displays a linear regression line with a 95% confidence interval and marginal density plots showing the distribution of ID1 expression (*x*-axis) and immune cell proportions (*y*-axis). Spearman correlation coefficients (r) and *p*-values are indicated within each panel.

As shown in Figure 7, ID1 expression exhibited significant positive correlations with several innate immune cell types, including neutrophils (r = 0.39, *p* = 1.2 × 10^−7^), resting mast cells (r = 0.19, *p* = 0.014), monocytes (r = 0.22, *p* = 0.0037), and activated dendritic cells (r = 0.42, *p* = 8.5 × 10^−9^). In contrast, negative correlations were observed with activated NK cells (r = −0.25, *p* = 0.00079), as well as with all three macrophage subtypes: M2 (r = −0.36, *p* = 1.7 × 10^−6^), M1 (r = −0.29, *p* = 0.00013), and M0 (r = −0.42, *p* = 1.2 × 10^−8^).

To provide an integrated overview, correlations across all 22 immune cell types were visualized in a bubble plot (Supplementary Figure S2), where dot size represents the magnitude of correlation (|r|) and color corresponds to statistical significance. The plot confirmed the key positive and negative relationships identified in the scatter plots and revealed additional subtle trends, such as mild positive associations with plasma cells and γδ T cells, and negative correlations with resting dendritic cells and regulatory T cells (Tregs). Statistically significant relationships (*p* < 0.05) were highlighted for clarity.

Overall, these analyses indicate that ID1 expression is closely associated with specific patterns of immune cell enrichment and depletion in AAA.

### 3.8. Single-Cell Validation of ID1 Expression in AAA

**Figure 8:**
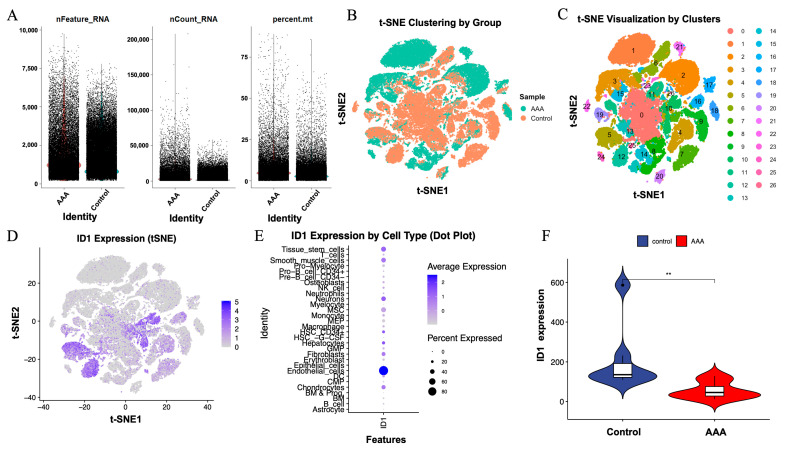
Single-cell RNA sequencing validation of ID1 expression in AAA and control samples. (**A**) Quality control metrics, including nFeature_RNA, nCount_RNA, and percent.mt, for cells derived from AAA and control tissues. Cells are colored by sample group, with AAA shown in red and control shown in blue. (**B**) t-SNE plot colored by sample group (AAA vs. control). (**C**) t-SNE visualization showing 26 transcriptionally defined cell clusters across all cells. (**D**) ID1 expression projected onto the t-SNE space, indicating cell-type-specific distribution. (**E**) Dot plot displaying ID1 expression across different cell types, where dot size represents the proportion of expressing cells and color denotes the average expression level. (**F**) Violin plot comparing ID1 expression levels between control and AAA samples. Statistical significance was determined using the Wilcoxon test (** *p* < 0.01).

To independently validate the expression pattern and cellular localization of ID1 in AAA, we analyzed the publicly available scRNA-seq dataset GSE226492. Quality control metrics, including the number of detected genes (nFeature_RNA), total transcript counts (nCount_RNA), and mitochondrial transcript proportion (percent.mt), were comparable between AAA and control samples, indicating consistent sequencing quality (Figure 8A).

t-SNE analysis revealed clear segregation of cells by sample type (Figure 8B) and identified 26 transcriptionally distinct clusters across all cells (Figure 8C). Visualization of ID1 expression within the t-SNE space showed enrichment in specific cellular regions (Figure 8D), indicating a cell-type-restricted expression pattern.

Dot plot analysis further demonstrated that ID1 was predominantly expressed in endothelial cells, followed by epithelial cells and fibroblasts, based on both average transcript abundance and the proportion of expressing cells (Figure 8E). Notably, ID1 expression was significantly reduced in AAA samples compared with controls (*p* < 0.01; Figure 8F), consistent with results obtained from bulk transcriptomic analyses.

Together, these single-cell results showed that ID1 was predominantly localized to vascular and stromal compartments and was downregulated in AAA tissues. This cell-type-specific reduction indicates that ID1-associated transcriptional changes are primarily observed in endothelial and fibroblast compartments, providing cellular context for downstream analyses.

## 4. Discussion

In this study, we conducted an integrative transcriptomic analysis to explore transcriptomic alterations associated with AAA and to prioritize candidate genes linked to disease pathogenesis. Through integration of differential expression analysis, WGCNA, and three machine learning–based feature selection algorithms (LASSO, RF, and SVM-RFE), we systematically prioritized 244 candidate genes derived from the GSE232911 dataset. Among these, ID1 and CYP4B1 were consistently identified across all models. However, CYP4B1 showed inconsistent expression patterns and classification performance between datasets, likely due to batch effects, patient heterogeneity, or tissue sampling differences, limiting its reproducibility. In contrast, ID1 showed reproducible downregulation and stable discriminative performance across datasets, supporting its prioritization for further biological investigation in AAA.

ID1 encodes a member of the inhibitor of DNA binding (ID) family of helix–loop–helix transcriptional regulators, which modulate gene expression by forming non-DNA-binding heterodimers with basic helix–loop–helix proteins, thereby preventing their binding to target DNA sequences [30]. It has established roles in angiogenesis, vascular remodeling, and mesenchymal cell differentiation, acting as an important contributor to endothelial stability and tissue repair [31,32,33,34,35]. In the cardiovascular system, ID1 is regulated by the TGF-β, BMP, and VEGF pathways and has been implicated in vascular homeostasis and anti-inflammatory signaling [36,37].

Given that AAA pathogenesis involves chronic inflammation, ECM degradation, and VSMC phenotypic modulation, the observed downregulation of ID1 implies potential involvement in these processes. Functional enrichment of ID1-associated genes highlighted pathways related to immune regulation, ECM organization, and cytokine-mediated signaling, aligning with established mechanisms of AAA progression. Although direct experimental evidence linking ID1 to specific inflammatory signaling cascades in AAA is limited, the known interactions between ID1 and TGF-β/BMP signaling pathways, which are functionally linked to inflammatory regulation, suggest a potential indirect association between ID1 and vascular inflammation [38,39]. Previous studies have shown that loss of ID1 enhances pro-inflammatory activation and endothelial dysfunction [40]. Because NF-κB activation drives expression of adhesion molecules such as VCAM-1 and ICAM-1 [41], reduced ID1 expression may be indirectly associated with vascular inflammation, a hypothesis that warrants experimental validation.

Beyond vascular structural regulation, members of the ID protein family are known modulators of immune balance. For instance, ID3 has been shown to promote regulatory T-cell (Treg) differentiation and maintain peripheral tolerance [42,43]. While ID3 has been extensively characterized, the immunomodulatory role of ID1 remains less defined. Our immune functional analyses provide preliminary insights into this aspect. Specifically, samples with low ID1 expression exhibited a pattern of selective immune remodeling rather than global immune activation. Adaptive immune pathways related to antigen presentation, dendritic cell activity, and T helper cell signaling were upregulated, whereas several innate immune components, including mast cells and neutrophils, were relatively suppressed. This immune profile suggests a shift toward adaptive immune predominance that may contribute to sustained inflammation within the aneurysmal wall.

Consistent with these findings, immune cell deconvolution using CIBERSORT revealed increased infiltration of M1 macrophages, γδ T cells, and memory B cells in samples with low ID1 expression, alongside reduced proportions of monocytes, neutrophils, and NK cells. Interestingly, despite reduced neutrophil abundance, neutrophil-associated functional scores were elevated, which may reflect enhanced activation on a per-cell basis or transient recruitment of hyperactivated subsets. Collectively, these observations are associated with specific patterns of immune cell redistribution and functional variation in AAA, although causal relationships cannot be inferred from the present analysis.

Single-cell transcriptomic validation further demonstrated that ID1 is predominantly expressed in endothelial and stromal fibroblast populations and significantly reduced in AAA tissues. This cell-type-specific distribution suggests that ID1 may be involved in local processes related to vascular integrity, ECM remodeling, and paracrine immune signaling. Previous studies have similarly shown that endothelial ID1 contributes to vascular protection in pulmonary hypertension by limiting inflammatory activation and promoting angiogenic balance [44]. Collectively, these findings suggest that ID1 may participate in vascular immunoregulatory processes across distinct contexts.

Experimental studies in animal models have highlighted an important role of ID1 in maintaining vascular homeostasis. Loss of ID1, especially in conjunction with ID3 deficiency, leads to impaired endothelial differentiation, vascular rarefaction, and tissue fibrosis [45,46,47]. Conditional deletion in endothelial or hematopoietic lineages triggers microvascular injury and activation of profibrotic TGF-β signaling, suggesting that ID1 may function through both cell-autonomous and intercellular mechanisms. These findings are consistent with our observation that ID1 is positioned at the intersection of vascular structure and immune regulation in AAA.

Despite these mechanistic insights, several limitations should be noted. First, the present analysis is computational and lacks in vitro or in vivo validation. Second, the scRNA-seq dataset comprised a limited number of samples and may not capture the full spectrum of disease heterogeneity. Third, both WGCNA and machine learning models are sensitive to data normalization and sample distribution, emphasizing the need for verification in larger, multicenter cohorts. Future studies should focus on experimental validation using gene perturbation models—such as CRISPR-mediated ID1 knockout or overexpression—to assess its causal role in AAA progression.

**Figure 9:**
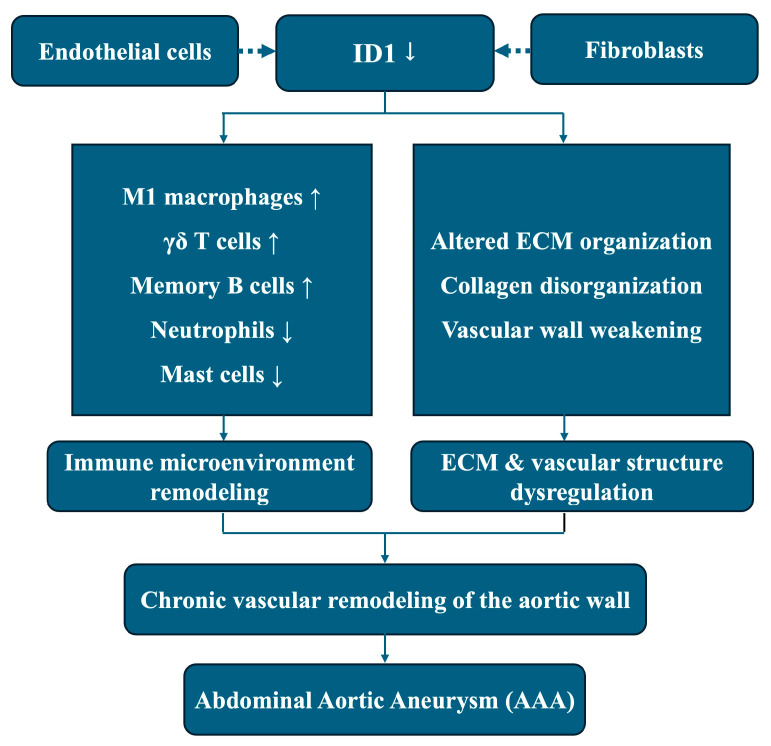
This schematic illustrates an association-based conceptual model derived from integrative transcriptomic analyses. Reduced ID1 expression, predominantly localized to endothelial cells and fibroblasts, is associated with immune microenvironment remodeling and ECM dysregulation in aneurysmal tissue, including altered immune cell composition and disrupted ECM organization. These coordinated transcriptomic alterations are linked to chronic vascular remodeling of the aortic wall and the development of AAA. Dashed arrows indicate transcriptomic associations or cell-type–specific localization rather than direct causal or regulatory relationships.

In summary, this integrative transcriptomic study identifies ID1 as a consistently downregulated gene associated with immune microenvironment remodeling and ECM–related transcriptional programs in AAA. By integrating bulk RNA sequencing, network analysis, immune deconvolution, machine learning, and single-cell RNA sequencing, our findings position ID1 as a candidate regulatory node linking vascular structural homeostasis and immune imbalance in aneurysmal tissue. These results are association-based and hypothesis-generating rather than indicative of direct molecular causality. An ID1-related schematic summarizing the proposed conceptual model is provided as Figure 9.

## 5. Conclusions

This study employed an integrative multi-omics and machine learning framework to characterize transcriptomic alterations associated with AAA. Across independent datasets and complementary analytical approaches, ID1 consistently emerged as a downregulated gene linked to immune microenvironment remodeling and ECM–related transcriptional programs in aneurysmal tissue. These findings position ID1 as a biologically plausible regulatory node connecting vascular structural integrity and immune imbalance in AAA. Collectively, this work underscores the utility of integrative transcriptomic analyses in complex vascular diseases and provides a focused foundation for future mechanistic investigations of ID1 in aneurysm pathobiology.

## Data Availability

The datasets analyzed in this study are available in the GEO database under accession numbers GSE232911, GSE183464, and GSE226492, and the processed data are available from the corresponding author upon reasonable request.

## Supplementary Materials

The following supporting information can be downloaded at: https://www.mdpi.com/article/10.3390/cimb48020156/s1.
