# Supplementary material for: Integrative Multi-Omics and Machine Learning Identify ID1 as a Candidate Gene Associated with Abdominal Aortic Aneurysm
Source: Curr Issues Mol Biol. 2026 Jan 30;48(2):156. doi: 10.3390/cimb48020156 (PMC12939636; doi:10.3390/cimb48020156)
Supplement: Supplementary file 1 [file cimb-48-00156-s001.zip › cimb-4066901-supplementary.pdf]

Supplementary Figure S1. Functional enrichment analysis of ID1-associated genes.

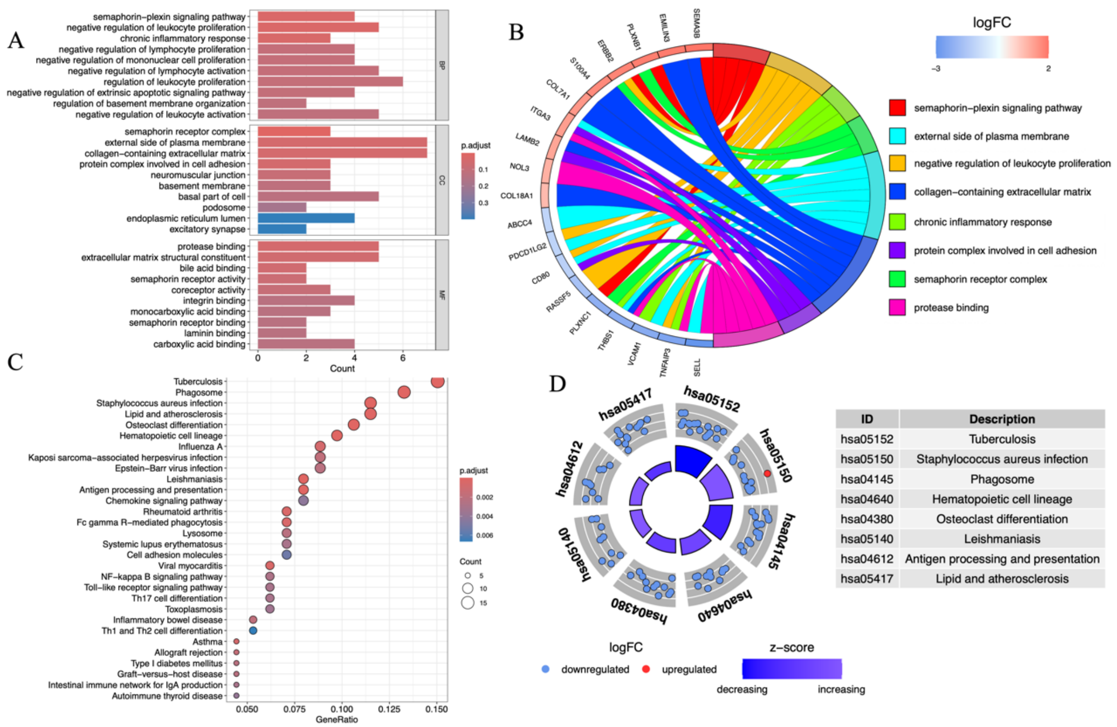

(A) Bar plot showing the top 10 significantly enriched GO terms across the biological process (BP), cellular component (CC), and molecular function (MF) categories. (B) Chord diagram illustrating the associations between representative genes and their corresponding GO terms. (C) Bubble plot of KEGG pathways, highlighting enrichment in inflammation-, immune-, and vascular remodeling–related pathways. (D) Circular plot depicting the interactions between selected genes and key KEGG pathways.

Supplementary Figure S2. Summary of correlations between ID1 expression and immune cell proportions in AAA.

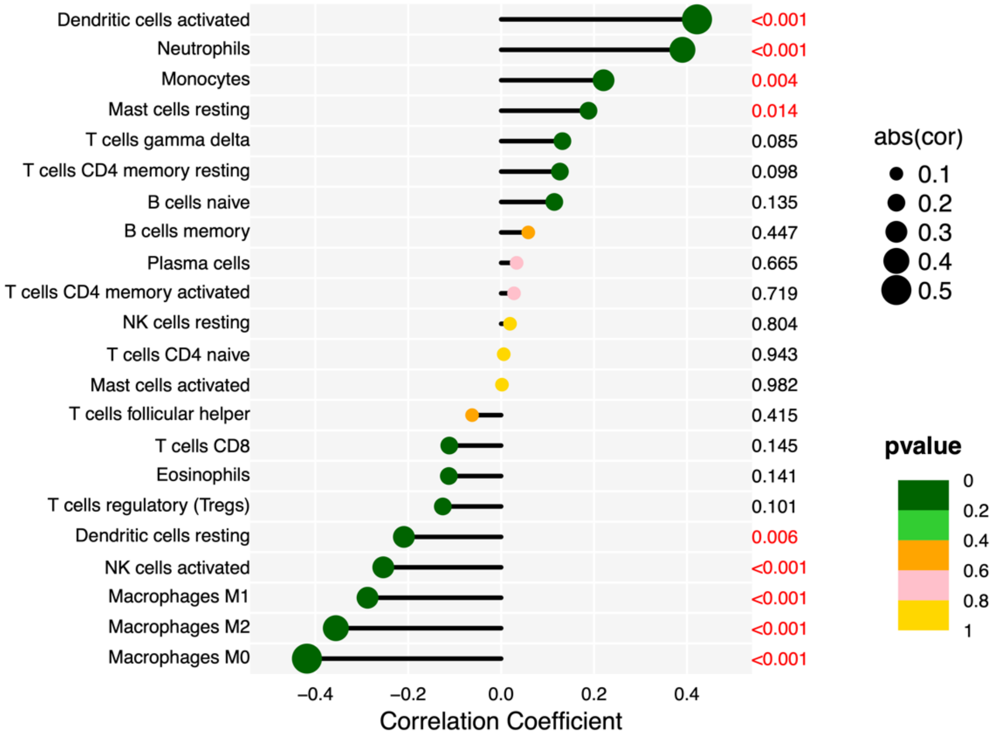

Bubble plot summarizing Spearman correlation coefficients between ID1 expression and 22 immune cell types in AAA samples. Dot size reflects correlation strength (|r|), while color indicates the corresponding P-value. Immune cell proportions were estimated using the CIBERSORT algorithm. Statistically significant correlations ($P < 0.05$) are highlighted for emphasis.
